# Role of Viral Infections in Testicular Cancer Etiology: Evidence From a Systematic Review and Meta-Analysis

**DOI:** 10.3389/fendo.2019.00355

**Published:** 2019-06-12

**Authors:** Andrea Garolla, Amerigo Vitagliano, Francesco Muscianisi, Umberto Valente, Marco Ghezzi, Alessandra Andrisani, Guido Ambrosini, Carlo Foresta

**Affiliations:** ^1^Unit of Andrology and Reproductive Medicine, Section of Endocrinology, Department of Medicine, Centre for Male Gamete Cryopreservation, University of Padova, Padova, Italy; ^2^Unit of Gynecology and Obstetrics, Department of Women and Children's Health, University of Padova, Padova, Italy

**Keywords:** viral infections, testicular cancer, human papillomavirus, human immunodeficiency virus, cytomegalovirus, Epstein–Barr virus, Parvovirus B-19

## Abstract

The most represented histotype of testicular cancer is the testicular germ-cell tumor (TGCT), both seminoma and non-seminoma. The pathogenesis of this cancer is poorly known. A possible causal relationship between viral infections and TGCTs was firstly evoked almost 40 years ago and is still a subject of debate. In the recent past, different authors have argued about a possible role of specific viruses in the development of TGCTs including human papillomavirus (HPV), Epstein–Barr virus (EBV), cytomegalovirus (CMV), Parvovirus B-19, and human immunodeficiency virus (HIV). The aim of this present review was to summarize, for each virus considered, the available evidence on the impact of viral infections on the risk of developing TGCTs. The review was reported following the Preferred Reporting Items for Systematic Reviews and Meta-Analyses (PRISMA) guidelines. We included all observational studies reported in English evaluating the correlations between viral infections (HPV, CMV, EBV, Parvovirus B19, and HIV) and TGCTs. The methodological quality of studies included in the meta-analysis was evaluated using a modified version of the “Newcastle–Ottawa Scale.” Meta-analyses were conducted using the “Generic inverse variance” method, where a pooled odds ratio (OR) was determined from the natural logarithm (LN) of the studies' individual OR [LN (OR)] and the 95% CI. A total of 20 studies (on 265,057 patients) were included in the review. Meta-analysis showed an association with TGCTs only for some of the explored viruses. In particular, no association was found for HPV, CMV, and Parvovirus B-19 infection (*p* = ns). Conversely, EBV and HIV infections were significantly associated with higher risk of developing TGCTs (OR 7.38, 95% CI 1.89–28.75, *p* = 0.004; OR 1.71, 95% CI 1.51–1.93, *p* < 0.00001). In conclusion, we found adequate evidence supporting an oncogenic effect of HIV and EBV on the human testis. Conversely, available data on HPV and TGCTs risk are conflicting and further studies are needed to draw firm conclusions. Finally, current evidence does not support an effect of CMV and Parvovirus B-19 on testicular carcinogenesis.

## Introduction

Testicular cancer (TC) is the most common solid tumor affecting males between 20 and 40 years old and accounting for approximately 1–1.5% of all cancers in men (1, 2). In the last decades, its incidence showed a progressive increase, particularly in some regions of Europe and Northern America (3–5). It is a real variegate cancer, characterized by several histological patterns, comprising germ-cell tumors and non-germ-cell tumors. The former group is the most common and it is further subdivided into two histologic subtypes, namely, seminomas and non-seminomas (6).

Many risk factors have been proposed for TGCTs (7, 8) including cryptorchidism, genetics, and substances of abuse (i.e., drugs, smoke, and hormones). In addition, it is well known that some testicular lesions mimicking a testicular tumor are due to infectious pathology, especially in immunosuppressed patients. Nevertheless, the possible causal relationship between viral infections and TGCTs is still a subject of debate.

Despite the exposure to some viruses that have been certainly associated to other cancer types in males [Epstein–Barr virus (EBV) for Burkitt lymphoma, human immunodeficiency virus (HIV) for Kaposi's sarcoma, hepatitis B virus (HBV) and hepatitis C virus (HCV) for hepatocellular carcinoma, and human papilloma virus (HPV) for penile, oropharyngeal, and anal cancers], few studies evaluated the possible implications of viral infection in the pathogenesis of TGCTs. Curiously, most of the viruses involved in sexually transmittable disorders have an age-related prevalence that coincides with that observed in TGCTs. Moreover, the characteristic long latency and persistence in the host of several viruses could induce a long-term dysregulation of the cell cycle able to induce the cancer development. Again, some studies demonstrated the prevalence of viral DNA/RNA directly in tissue specimens from testicular cancer (9–11). Finally, it has been reported that EBV, as well as other DNA viruses, encodes a protein able to inactivate p53, a mechanism that is able to reduce apoptosis in tumor cells (10, 12).

In this meta-analysis, we aimed to summarize the whole body of literature exploring the correlation between TGCTs and viral infections by HPV, HIV, cytomegalovirus (CMV), EBV, and Parvovirus B-19, with the purpose of clarifying the possible role of these viruses in the pathogenesis of this condition.

## Materials and Methods

### Study Design

This is a systematic review and meta-analysis of published data. The review was reported following the Preferred Reporting Items for Systematic Reviews and Meta-Analyses (PRISMA) guidelines (13).

### Ethical Approval

As this study was a systematic review and meta-analysis of published data, formal ethical approval was not required.

### Search Strategy

Electronic databases (Sciencedirect, Medline, Scopus, Embase, the Cochrane library, Clinicaltrials.gov, EU Clinical Trials Register and World Health Organization International Clinical Trials Registry) were searched until 1st February 2019 (without date restriction).

Key search terms were as follows: virus OR viral infection OR viral disease OR human papillomavirus OR HPV OR Cytomegalovirus OR CMV OR Epstein–Barr virus OR EBV OR Parvovirus B19 OR human immunodeficiency virus OR HIV OR acquired immunodeficiency syndrome OR AIDS AND testicular cancer OR testicular neoplasm OR testicular tumor. The electronic search and the eligibility of the studies were independently assessed by two of the authors (AG and FM).

### Inclusion Criteria

We included all studies evaluating the correlations between viral infections (i.e., HPV, CMV, EBV, Parvovirus B19, and HIV) and TGCTs. All observational studies (retrospective and prospective cohort studies, case and control series) reported in English were eligible. Testicular cancer was defined as the demonstration of testicular cancer cells at histopathological examination.

### Study Selection and Data Extraction

Two authors (AG and AV) independently assessed the inclusion criteria and study selection. Disagreements were discussed with a third reviewer (CF).

Data extraction was performed by five independent investigators (AA, GA, FM, MG, and UV). When studies involved a subgroup of patients considered negligible for the endpoints of meta-analysis (e.g., patients affected by non-testicular cancer), the authors provided only a qualitative data extraction. A manual search of reference lists of studies was performed to avoid missing relevant publications. One author (AV) reviewed the selection and data extraction process. The results were then compared, and any disagreement was discussed and resolved by consensus.

### Risk of Bias

Two reviewers (AV and AG) independently judged the methodological quality of studies included in the meta-analysis using a modified version of the “Newcastle–Ottawa Scale” (14). Quality of studies was evaluated in five different domains: “*sample representativeness*,” “*sampling technique*,” “*ascertainment of viral infection*,” “*quality of description of the population and confounders*,” and “*incomplete data on cancer histology*” (Table S1). According to the total number of points assigned, each study was judged to be at low risk of bias (≥3 points) or high risk of bias (<3 points). Any discrepancies concerning the author's judgments were referred to a third reviewer (CF) and resolved by consensus.

### Statistical Analysis

Odds ratios (ORs) and proportions were calculated with MedCalc 18.5 (MedCalc Software, Seoul, 158-051, Korea). For meta-analysis, Review Manager (RevMan) Version 5.1 (The Nordic Cochrane Centre, The Cochrane Collaboration, 2011) was used. Statistical analysis was conducted using the “Generic inverse variance” method, where a pooled OR was determined from the natural logarithm (LN) of the studies' individual OR [LN (OR)] and the 95% CI. The SE for the LN (OR) was calculated from the 95% CI using the formula: SE = [LN (upper CI limit) – LN (lower CI limit)]/3.92, according to the Cochrane Reviewers' handbook (15). Statistical heterogeneity was assessed by *I*^2^ statistics. The pooled estimates were reported graphically with Forest plots. Meta-analyses were conducted separately for each virus (HPV, EBV, CMV, Parvovirus B-19, and HIV). Sources of statistical heterogeneity were investigated by subgroups and sensitivity analyses (by serially excluding each study or study subgroups basing on methodological quality judgments).

## Results

### Study Selection

**Figure 1:**
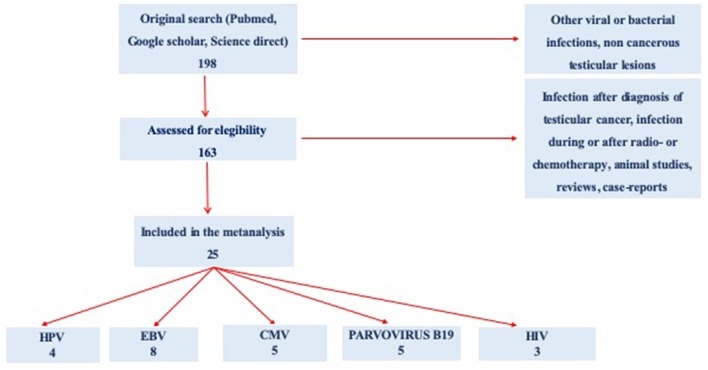
Preferred Reporting Items for Systematic Reviews and Meta-Analyses **(**PRISMA) flow diagram of the review study on literature analysis for viral infection in subjects with testicular cancer. Some of the studies evaluated more than a virus.

Starting from 198 selected abstracts, we evaluated 163 full texts regarding infection of interest in subjects with testicular cancer. Because of confounding conditions (infection after diagnosis of TC infection during or after radio and/or chemotherapy, animal studies, reviews, and case reports), 138 studies were excluded. Finally, a total of 25 studies were included in the present meta-analysis: 4 for HPV, 8 for EBV, 5 for CMV, 5 for Parvovirus B19, and 3 for HIV (Figure 1).

### Included Studies

Characteristics of included studies are summarized in Table 1. We included in the review a total of 19 studies. Four studies provided information for two viruses (19, 21–23) and one for three (24) viruses, reaching the number of 25. Studies embedded a total of 285,878 subjects. Most of the studies were case–control except for one case series, for both HPV and EBV (19) and one survey for HPV (18). All studies on HIV were cohort studies (31–33). Different techniques to diagnose viral infections were used. Most studies used PCR or detection of serum antibodies. Six studies used immune-histochemistry (IHC), four used *in situ* hybridization (ISH), one used fluorescent *in situ* hybridization (FISH), and one used immunofluorescent staining (IFS).

**Table 1:** General features of included studies.

**Virus**	**References**	**Country**	**Setting**	**Population**	**Method**	**Measure**	**Result (95% CI)**
HPV	Bertazzoni et al. (16)	Italy	Case–control	Cases 61 Controls 23	PCR	Percentage	0% Cases 0% Controls
HPV	Garolla et al. (17)	Italy	Case–control	Cases 155 Controls 84	PCR and FISH	Percentage	9.7% Cases 2.4% Controls
HPV	Strickler et al. (18)	USA	Survey	Total 87 (TC 39)	ELISA	Percentage	5% of TC
HPV	Rajpert-De Meyts et al. (19)	Denmark	Case series	Cases 19 Controls 1	PCR and IHC	Number of cases	0% Cases 0% Controls
EBV	Moss et al. (20)	USA	Case–control	Cases 173 Controls 217	Interview	OR	0.6 (0.3–1.1)
EBV	Algood et al. (21)	USA	Case–control	Cases 56 Controls 30	Serology	Percentage	80% Cases 30% Controls
EBV	Akre et al. (22)	Norway	Case–control	Cases 81 Controls 242	Serology	OR	2.74 (0.62–12.12)
EBV	Shimkage et al. (10)	Japan	Case–control	Cases 27 Controls 25	PCR, IHC, and IFS	Percentage	100% Cases 0% Controls
EBV	Heinzer et al. (23)	Germany	Case–control	Cases 53 Controls 51	Serum antibodies	OR	6.93 (0.8–59.8)
EBV	Gray et al. (24)	Switzerland	Case–control	Cases 39 Controls 12	PCR	Percentage	0% Cases 0% Controls
EBV	Rajpert-De Meyts et al. (19)	Denmark	Case series	Cases 19 Controls 1	PCR, IHC, and ISH non-radioactive	Proportion	6/19
EBV	Fend et al. (25)	Austria	Case–control	Cases 32 Controls 5	PCR and ISH non-radioactive	Percentage	12.5% Cases 0% Controls
CMV	Mueller et al. (26)	Sweden	Case–control	Cases 117 Controls 100	Serology	R.R.	2.0 (1.1–3.6)
CMV	Akre et al. (22)	Norway	Case–control	Cases 81 Controls 242	Serology	OR	1.08 (0.6–1.94)
CMV	Algood et al. (21)	USA	Case–control	Cases 56 Controls 10	Serology	Percentage	50% Cases 10% Controls
CMV	Heinzer et al. (23)	Germany	Case–control	Cases 47 Controls 47	ISH and Serum antibodies	Percentage	4.2% Cases 0% Controls
CMV	Gray et al. (24)	Switzerland	Case–control	Cases 39 Controls 12	PCR	Percentage	0% Cases 0% Controls
Parvo B19	Polzc et al. (27)	USA	Case–control	Cases 23 Controls 7	PCR and IHC	Percentage	73.9% Cases 85.7% Controls
Parvo B19	Gray et al. (24)	Switzerland	Case–control	Cases 39 Controls 12	PCR	Percentage	85% Cases 0% Controls
Parvo B19	Ergunay et al. (28)	Turkey	Case–control	Cases 56 Controls 66	PCR	Percentage	5.4% Cases 0% Controls
Parvo B19	Tolfvenstam et al. (29)	Norway	Case–control	Cases 77 Controls 238	PCR, Serology and IHC	OR	1.03 (0.6–1.77)
Parvo B19	Diss et al. (30)	UK	Case–control	Cases 20 Controls 10	PCR and IHC	OR	1.01 (0.59–1.72)
HIV	Dihr et al. (31)	India	Cohort	Total 251 (5 TC)	Serology	PIR	2.5 (1.04–6.05)
HIV	Goedert et al. (32)	USA	Cohort	Total 268.950 (217 TC)	Serology	SIR	1.7 (1.5–1.9)
HIV	Grulich et al. (33)	Australia	Cohort	Total 13.067 (10 TC)	Serology	SIR	1.46 (0.7–2.69)

*FISH, fluorescent in situ hybridization; IHC, immune-histochemistry; IFS, immunofluorescent staining; ELISA, enzyme-linked immunosorbent assay; PIR, proportioned incidence ratio*.

### Assessment of the Risk of Study Bias

In Table 2, the criteria used to assess the risk of study bias are reported.

– *Sample representativeness:* All but six studies (10, 19, 24, 25, 27, 30) were judged at low risk of bias for sample representativeness.
– *Sampling technique*: Eleven studies (16, 17, 20–22, 24, 26, 29, 31–33) had adequate sampling strategy (random or consecutive). Other studies did not provide data.
– *Ascertainment of viral infection:* One study (20) was judged at high risk of bias because the diagnosis of viral infection was based on self-administered questionnaires. The remaining studies were at low risk of bias.
– *Quality of description of the population and confounders:* Only three studies were considered at low risk of bias (17, 20, 28). Other studies did not provide adequate description of the study population and/or confounders.
– *Incomplete data on histology*: All but two studies (17, 31) provided adequate data on cancer histotypes.
– *Overall study quality*: In summary, pooling of scores for each domain resulted in five studies to be at high risk of bias (10, 19, 25, 27, 30). The remaining studies were at low risk of bias (16–18, 20–24, 26, 28, 29, 31–33).

**Table 2:** Authors' judgment of study quality according to the “Modified Newcastle–Ottawa Risk of Bias Scoring System.”

**References**	**Sample representativeness**	**Sampling technique**	**Diagnostic accuracy**	**Confounders description**	**Cancer histology**	**Total score**	**Risk of bias**
Bertazzoni et al. (16)	^*^	^*^	^*^		^*^	4	LOW
Garolla et al. (17)	^*^	^*^	^*^	^*^		4	LOW
Polzc et al. (27)			^*^		^*^	2	HIGH
Moss et al. (20)	^*^	^*^		^*^	^*^	4	LOW
Algood et al. (21)	^*^	^*^	^*^		^*^	4	LOW
Akre et al. (22)	^*^	^*^	^*^		^*^	4	LOW
Shimkage et al. (10)			^*^		^*^	2	HIGH
Heinzer et al. (23)	^*^		^*^		^*^	3	LOW
Gray et al. (24)		^*^	^*^		^*^	3	LOW
Rajpert-De Meyts et al. (19)			^*^		^*^	2	HIGH
Fend et al. (25)			^*^		^*^	2	HIGH
Mueller et al. (26)	^*^	^*^	^*^		^*^	4	LOW
Ergunay et al. (28)	^*^		^*^	^*^	^*^	4	LOW
Tolfvenstam et al. (29)	^*^	^*^	^*^		^*^	4	LOW
Diss et al. (30)			^*^		^*^	2	HIGH
Strickler et al. (18)	^*^		^*^		^*^	3	LOW
Dihr et al. (31)	^*^	^*^	^*^			3	LOW
Goedert et al. (32)	^*^	^*^	^*^		^*^	4	LOW
Grulich et al. (33)	^*^	^*^	^*^		^*^	4	LOW

The symbol

* *indicate the presence of the criterion considered in the table*.

### Synthesis of Results

#### Human Papilloma Virus

**Figure 2:**
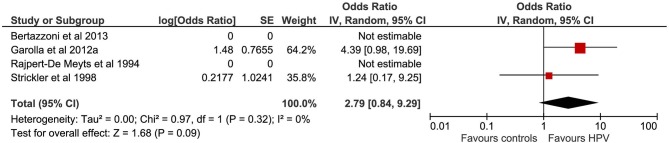
Forest plot: Human papilloma virus (HPV) and testicular cancer.

A total of four studies evaluated the correlation between HPV infection and TGCTs. The pooled sample of patients analyzed was 430, of whom 274 were affected by TGCTs and 156 were healthy controls (Figure 2). In two studies, the search for HPV was conducted on histological sections from testicular tissue using PCR (16, 19). In the remaining two studies, the diagnosis of HPV infection was achieved by detection of serum antibodies (18) and by evaluating sperm infection by FISH and PCR (17).

Pooling of results did not show an association between HPV infection and TGCTs (OR 2.79, 95% CI 0.84–9.29, *p* = 0.09, *I*^2^ = 0%). Sensitivity analysis did not provide statistical changes to aggregate results. Subgroup analysis was not feasible.

#### Epstein–Barr Virus

**Figure 3:**
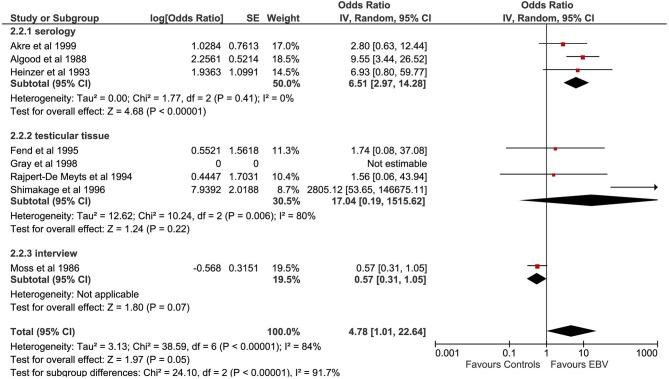
Forest plot: Epstein–Barr virus (EBV) and testicular cancer.

A total of eight studies evaluated the correlation between EBV infection and TGCTs. The pooled sample of patients analyzed was 1,063, of whom 480 were affected by TGCTs and 583 were controls (Figure 3). In four studies, the search for EBV was conducted on histological sections from testicular tissue by using ISH, IFS, and PCR (10); PCR (24); and PCR, IHC, and on-radioactive ISH (19, 25). In a single study, the history of EBV infection was evaluated by telephone interview (20). In the remaining three studies, EBV diagnosis was achieved by detection of serum antibodies (21–23).

Pooling of results did not show an association between EBV infection and TGCTs (OR 4.78, 95% CI 1.01–22.64, *p* = 0.05), with high degree of statistical heterogeneity (*I*^2^ = 84%). The exclusion of the study by Moss et al. from meta-analysis resulted in a significant association between EBV and TGCTs (OR 7.38, 95% CI 1.89–28.75, *p* = 0.004, *I*^2^ = 59%). Subgroup analysis based on the methods for EBV determination (serology vs. testicular tissue analysis vs. interview) found a significantly higher risk of TGCTs in those patients with a positive serology (test for subgroup differences: χ^2^ = 24.1, *p* < 0.00001). The proportion of seminomas among EBV+ patients (at serology) with a diagnosis of TGCTs was 51.69% (95% CI 44.01–59.32%).

#### Cytomegalovirus

**Figure 4:**
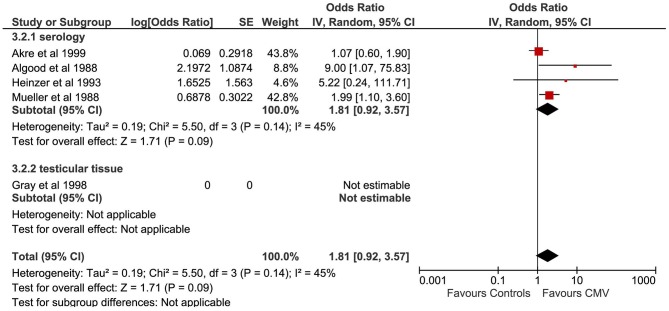
Forest plot: Cytomegalovirus (CMV) and testicular cancer.

A total of five studies evaluated the correlation between CMV infection and TGCTs (Figure 4). The pooled sample of patients analyzed was 751, of whom 340 were affected by TGCTs and 411 were controls. In a single study, the search for CMV was conducted on histological sections from testicular tissue by PCR (24). In four studies, CMV diagnosis was achieved by detection of serum antibodies (22, 23, 26). One study used indirect immunofluorescence assay (21).

Pooling of results did not show an association between CMV infection and TGCTs (OR 1.81, 95% CI 0.92–3.57, *p* = 0.09), with moderate statistical heterogeneity (*I*^2^ = 45%). The exclusion of the study by Akre et al. from the meta-analysis resulted in a significant association between CMV and TGCTs (OR 2.38, 95% CI 1.24–4.53, *p* = 0.009) and reduced the heterogeneity (*I*^2^ = 4%). The proportion of seminomas among CMV+ patients (at serology) with a diagnosis of TGCTs was 50.47% (95% CI 42.95–57.97%). Subgroup analysis was not feasible.

#### Parvovirus B-19

**Figure 5:**
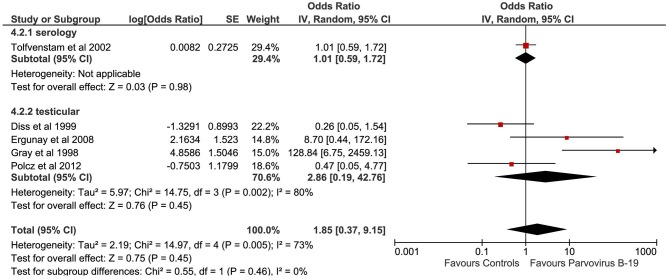
Forest plot: Parvovirus B-19 and testicular cancer.

A total of five studies evaluated the correlation between Parvovirus B-19 infection and TGCTs (Figure 5). The pooled sample of patients analyzed was 548, of whom 215 were affected by TGCTs and 333 were controls. In a single study, the search for Parvovirus B-19 was conducted by detection of serum antibodies (29). In four studies, Parvovirus B-19 diagnosis was achieved by analyzing samples of testicular tissue by PCR and IHC (27, 30) or only PCR (24, 28).

Pooling of results did not show an association between Parvovirus B-19 infection and TGCTs (OR 1.85, 95% CI 0.37–9.15, *p* = 0.45), with substantial statistical heterogeneity (*I*^2^ = 73%). The serial exclusion of each single study through sensitivity analysis as well as subgroup analysis did not modify the results of the primary analysis.

### Human Immunodeficiency Virus

**Figure 6:**
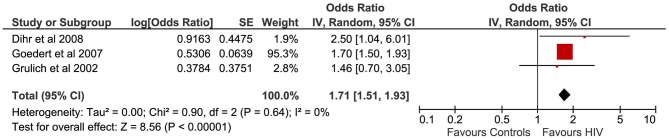
Forest plot: Human immunodeficiency virus (HIV) and testicular cancer.

A total number of three cohort studies (31–33) evaluated the correlation between HIV infection and testicular cancer (Figure 6). The pooled sample of patients analyzed was 282,268 HIV+, of whom 232 were affected by TGCTs. In all patients, the diagnosis of HIV was confirmed by serology.

Pooling of results showed a significant association between HIV infection and TGCTs (OR 1.71, 95% CI 1.51–1.93, *p* < 0.00001), with low heterogeneity (*I*^2^ = 0%). The serial exclusion of each single study through sensitivity analysis did not modify the results of the primary analysis. The proportion of seminomas among HIV+ patients with a diagnosis of TGCTs was 75.17% (95% CI 69.01–80.66%).

## Discussion

Testicular cancer is the most common neoplasm affecting males between 20 and 40 years old and accounting for approximately 1–1.5% of all cancers in men (1, 2). It embraces several histotypes of cancer, classified into the two main groups of seminomas and non-seminomas by the World Health Organization (6).

The pathogenesis of TGCTs is poorly known (34). Genetic factors play an important role in the development of this disease, as demonstrated by the modified expression of specific genes in testicular cancer cells (34, 35). Moreover, the exposition to different environmental agents, such as pesticides and non-steroidal estrogens (i.e., diethylstilbestrol), can increase the risk of developing this neoplasm (35, 36). Additional risk factors correlated to the onset of TGCTs are cryptorchidism, Klinefelter's syndrome, congenital abnormalities, and infertility (2, 8).

Recent efforts in oncological and virological research have brought to light the oncogenic potential of different virus species (37). It is now estimated that ~10% of worldwide cancers are attributable to viral infections, with the vast majority (85%) occurring in the developing world (37, 38). A possible causal relationship between viral infections and TGCTs was firstly evoked almost 40 years ago. Newell et al. (39) postulated a “viral theory” starting from the evidence of a similar geographical and age distribution of TGCTs and classical Hodgkin's lymphoma. In Hodgkin's lymphoma, the malignant Reed–Sternberg cells display a monoclonal profile where EBV DNA and RNA have been clearly identified (40, 41). Differently, data about EBV DNA and RNA within TGCT cells are few and the etiopathogenetic role of EBV in testicular carcinogenesis is still a matter of debate.

In the recent past, different authors have argued about a possible role of other oncogenic viruses in the development of TGCTs, including CMV, HIV, HPV, and Parvovirus B-19. The aim of this present review was to summarize the available evidence on the impact of viral infections on the risk of developing TGCTs.

### Main Findings and Interpretation

A total of 19 studies (10, 16–33) were included in this present systematic review and meta-analysis.

The correlation between HPV infection and TGCTs was evaluated by four studies on 430 patients (16–18, 31). Statistical analysis failed to demonstrate a statistical correlation between HPV infection and increased risk of TGCTs (*p* = 0.09). Notably, there was high between-studies heterogeneity in terms of methodology, potentially limiting drawing firm conclusions from the data.

HPV is one of the most common sexually transmitted viruses (42). It is particularly common in a young sexually active population and its prevalence is closely related to sexual behavior (3). During infection, HPV gains access to the interior of the cells, exerting a direct control on the proliferation and apoptosis of host cells (43). Once inside the cell, HPV DNA can transition from an episomal to a host genome integrated form, thus regulating cell genome transcription. Two specific HPV genes, namely, E6 and E7, are highly conserved among oncogenic HPV genotypes (44, 45). These genes can promote cellular transformation and alter the pathways related to the immune response, leading to carcinogenesis in a plethora of human tissues including vulva, vagina, penis, anus, head, neck, and oropharyngeal cavity (46, 47). In the testis, HPV is capable of directly infecting the male gametes, resulting in reduced fertility due to increased sperm DNA fragmentation and aneuploidy. It is thought that HPV is attached to the spermatozoa in two distinct sites along the equatorial region of the spermatozoon's head, similarly to other viruses infecting the sperm (42, 48).

Nevertheless, concurrently with new insights about infertility causes and treatments (49, 50), we must stress that the majority of recent studies on HPV in males have focused on the impact of viral infection on fertility, oocyte fertilization rate, and miscarriage rate in assisted reproduction technologies (ARTs) (51, 52). Conversely, the data on the association between HPV and TGCTs are scanty. Given the well-known oncogenic potential of HPV and considering its tropism for testicular tissue, the role of this virus in testicular carcinogenesis cannot be excluded. Future good-quality evidence is still needed to clarify the issue.

The association between EBV infection and TGCTs was investigated by eight studies on 1,063 patients (10, 19–25). While pooling of results from all studies did not show an association between EBV infection and TGCTs (*p* = 0.05), the exclusion of a single study from meta-analysis (20) resulted in a significant association between EBV and TGCTs (*p* = 0.004). Importantly, the study by Moss et al. (20) was at high risk of detection bias (i.e., the infection was investigated through a telephone interview), potentially distorting the final effect estimates from meta-analysis. Additionally, subgroup analysis (based on the methods for EBV determination) found a significant higher risk of TGCTs in those patients with a positive serology (*p* < 0.00001), further confirming the association between history of EBV infection and TGCTs onset.

EBV was the first virus shown to cause cancer in humans (53). Besides the well-known association between EBV and Burkitt lymphoma (discovered by Michael Anthony Epstein and Yvonne Barr in 1964) (54), this virus was found to be associated with many other lymphoid, epithelial, and mesenchymal cancers (55). EBV can promote carcinogenesis in both immune-competent hosts and immune-compromised patients (i.e., those who have undergone organ transplantation or who are under immune-suppressive treatments) (53, 56). The mechanisms of EBV-induced carcinogenesis rely on extensive methylation of the host genome, which promotes viral propagation and cellular transformation. The most common oncogenic DNA modifications associated with EBV are phosphatidylinositol-4,5-bisphosphate 3-kinase catalytic subunit alpha (PIK3CA) mutations, extreme DNA hypermethylation, and amplification of the Janus activated kinase2 (JAK2) (57, 58).

Regarding EBV and TGCTs, there is an epidemiological correlation between these two entities. The incidence of infectious mononucleosis (including EBV-correlated orchitis) is higher in Europe and North America, similar to that of TGCTs. Interestingly, both infectious mononucleosis and TGCTs mainly occur in adolescents, suggesting that testicular differentiation is a factor increasing the susceptibility for both EBV infection and testicular carcinogenesis. (10) Moreover, nasopharyngeal carcinoma (which is linked to EBV) and TGCTs have some common characteristics, including age peak incidences in adolescents and chemoresponsiveness to cisplatin (59). Therefore, this present review found adequate evidence supporting a role for EBV in TGCTs development.

The correlation between CMV infection and TGCTs was investigated by five studies, including 751 patients (19, 21–24, 26). While pooling of results did not show an association between CMV infection and TGCTs (*p* = 0.09), the exclusion of a single study (22) from meta-analysis resulted in a significant association between CMV and cancer (*p* = 0.009). Notably, the methodological quality of the study by Akre et al. (22) was fair. Therefore, based on available data, the correlation between CMV and TGCTs cannot be sustained.

CMV is a ubiquitous herpes virus that leads to a lifelong persistence (60). The prevalence of CMV infection is extremely high in the general adult population (from 50 to 100%) and the virus is not considered to be oncogenic (61). Probably, due to the high prevalence of the infection, a larger sample of patients would be required to show (or repudiate) any correlation between CMV and TGCTs.

Actually, even if CMV can lead to dramatic complications in immunocompromised individuals, murine experiments repeatedly failed to demonstrate a clear ongogenic activity for this virus (62, 63). In this regard, some authors postulated that CMV may contribute to oncogenesis by “hit-and-run” mechanisms, namely, by inducing human cell transformation and successively disappearing by malignant cell clones (64, 65). However, this theory is not adequately supported by scientific data and the oncogenic potential of CMV is still obscure.

A total of five studies (on 548 patients) evaluated the correlation between Parvovirus B-19 infection and TGCTs (24, 27–30). Pooling of results failed to demonstrate an association between Parvovirus B-19 infection and TGCTs (*p* = 0.45). Additionally, subgroup and sensitivity analyses did not modify the results of the primary analysis, confirming their robustness. Parvovirus B-19 is the only parvovirus known to be pathogenic for humans (66). The virus exhibits a particular tropism for erythroid cells and can rarely cause dramatic complications in humans (67, 68). In immunocompetent hosts, the virus can cause acute, generally self-limiting clinical manifestations including the fifth disease in children and acute polyarthritis in adults (66). In immunosuppressed hosts (including pregnant women), Parvovirus B-19 may cause severe complications including glomerulonephritis, vasculitis, peripheral neuropathies, myocarditis, fulminant hepatic failure, and aplastic anemia (69, 70). There is no robust evidence supporting the role of this virus in human cells oncogenesis, even if a recent study showed a possible correlation with thyroid cancer (71). However, available data do not support the role of Parvovirus B-19 in the etiology of TGCTs.

Three studies, performed on 282,268 patients, evaluated the correlation between HIV infection and TGCTs (31–33), showing a significant association between these two entities (*p* < 0.00001). The results were robust and displayed a low statistical heterogeneity (*I*^2^ = 0%).

The correlation between HIV-induced immunodeficiency and increased cancer risk has long been known (72). In immunocompetent people, the immune system has the ability to suppress oncogenic viruses and exert a continuous surveillance for malignant cells. These biological functions can fail when the immune system is impaired by HIV infection (73, 74). Therefore, HIV infection may promote testicular carcinogenesis mainly through indirect effects on the regulation of cell proliferation and apoptosis (72). Conversely, a direct effect of HIV on proto-oncogen expression in the human testis has not been demonstrated.

Interestingly, the majority of men dying of AIDS have hypospermatogenesis, spermatogenic arrest, or a Sertoli-cell-only testicular histology (75, 76). These histological changes are typically found in patients with TGCTs, supporting the theory that men with HIV may display a premalignant testicular atrophy. This premalignant condition may be due to the general debilitating effects of HIV rather than due to specific HIV-related mechanisms. To support this hypothesis, those patients effectively treated with antiretroviral drugs have a decreased incidence of testicular atrophy and TGCTs (77, 78). Therefore, we can conclude that HIV is associated with a significant higher risk of TGCTs, but effective antiretroviral therapy may considerably attenuate the risk of suffering from this condition.

### Strength and Limitations

The present meta-analysis comprehensively evaluates the impact of viral infection on TGCTs risk. We planned sensitivity and subgroup analysis in order to reduce bias related to study heterogeneity. Moreover, we created a modified Newcastle–Ottawa scoring system (*ad hoc*) in order to provide a methodological quality judgment for each study that may help readers in a proper interpretation of the study findings. However, our results are considerably limited by the small number of patients included in specific comparisons, heterogeneity in the study designs and methods, poor methodological quality of some studies (the majority were retrospective studies), and some concerns about the ascertainment of viral infection. In particular, while some studies used sensitive techniques to test viral infections, other studies, like those ones on HIV, based their results on the detection of serum antibodies. Moreover, only few studies reported the presence of other risk factors for the development of testicular cancer. Therefore, even if a relationship between specific viruses and testicular cancer was detected by the present meta-analysis, causation cannot be established.

## Conclusions

We found a possible correlation between specific viruses and testicular cancer, but the evidence was insufficient to establish causality. The correlation between HIV and increased risk of TGCTs is supported by good-quality evidence despite being based on serum antibody titers. Similarly, the evidence suggesting a link between EBV and TGCTs is fair.

Regarding the correlation between CMV and TGCTs, available data are conflicting and further studies are needed to draw firm conclusions. Moreover, poor evidence supports the lack of correlation between Parvovirus B-19 and a meaningful risk of TGCTs. Finally, data about the possible relationship between HPV and TGCTs are inconsistent, but its oncogenic potential for male gonadal tissue cannot be excluded; thus, future good-quality studies are warranted.

## Data Availability

No datasets were generated or analyzed for this study.

## Author Contributions

FM, UV, MG, AA, and GA performed the medline for article search. A manual search of reference lists of studies was performed to avoid missing relevant publications by FM. AG and AV independently assessed the inclusion criteria and study selection. Disagreements were discussed with the third reviewer CF. Data extraction was performed by two independent investigators AG and FM. AV reviewed the selection and data extraction process. The results were then compared, and any disagreement discussed and resolved by consensus of all authors. The draft was written by FM, AV, and AG.

### Conflict of Interest Statement

The authors declare that the research was conducted in the absence of any commercial or financial relationships that could be construed as a potential conflict of interest.

## Supplementary Material

Table S1 Risk of bias evaluation. Quality of studies included in the meta-analysis was evaluated using five different domains through a modified version of the “Newcastle–Ottawa Scale” (14).
