# Supplementary material for: Role of Viral Infections in Testicular Cancer Etiology: Evidence From a Systematic Review and Meta-Analysis
Source: Front Endocrinol (Lausanne). 2019 Jun 12;10:355. doi: 10.3389/fendo.2019.00355 (PMC6584824; doi:10.3389/fendo.2019.00355)
Supplement: Table S1 — Risk of bias evaluation. Quality of studies included in the meta-analysis was evaluated using five different domains through a modified version of the “Newcastle–Ottawa Scale” (14). [file Table_1.docx]

| **SAMPLE REPRESENTATIVENESS** |
| --- |
| ***1 point:*** sample size was grater or equal of 60 participants and rate exclusion was lower than 20% |
| ***0 point:*** sample size was less than 60 participants or exclusion rate was higher than 20% |
| **SAMPLING TECHNIQUE** |
| ***1 point:*** patients recruited consecutively or randomly (randomization criteria clarified) |
| ***0 point:*** potential convenience sampling or unspecified sampling technique |
| **DIAGNOSTIC ACCURACY** |
| ***1 point:*** highly specific laboratory techniques (serology, viral DNA/RNA detection, tissue analysis) |
| ***0 point:*** low quality techniques of viral detection (interview or questionnaire) |
| **CONFOUNDERS DESCRIPTION** |
| ***1 point:*** the study reported a clear description of population (age, possible confounders as cryptorchidism, genetic causes, drugs or substances of abuse) |
| ***0 point:*** the study did not report a clear description of population (incompletely or no reported confounders) |
| **CANCER HISTOLOGY** |
| ***1 point:*** clear description of cancer histology (germ cell or non germ cell cancer, seminoma and non seminoma) |
| ***0 point:*** no description of cancer histology was reported |

**Table S1.**
